# Violence risk prediction in mental health inpatient settings using the Dynamic Appraisal of Situational Aggression

**DOI:** 10.3389/fpsyt.2024.1460332

**Published:** 2024-12-10

**Authors:** Mario Moscovici, Farhat Farrokhi, Lavanya Vangala, Alexander I. F. Simpson, Paul Kurdyak, Roland M. Jones

**Affiliations:** ^1^ Forensic Psychiatry Division, The Centre for Addiction and Mental Health, Toronto, ON, Canada; ^2^ Department of Psychiatry, University of Toronto, Toronto, ON, Canada; ^3^ General and Health Systems Psychiatry Division, Centre for Addiction and Mental Health, Toronto, ON, Canada

**Keywords:** aggression, inpatient violence, risk assessment, prevention, forensic-psychiatric practice, acute psychiatric admission, old age psychiatry, schizophrenia

## Abstract

**Introduction/Background:**

Aggression and violence are common problems in healthcare settings and affects both patients and healthcare staff. The Dynamic Appraisal of Situational Aggression (DASA) is an assessment tool to guide assessment for short term risk in inpatient settings. There have been no large-scale studies examining the performance of the DASA across different clinical settings. Our objective is to examine the performance of the DASA using a large longitudinal patient sample on different clinical units. A secondary objective was to examine alterative risk categories of the DASA.

**Methods:**

All consecutive mental health hospital admissions to a large hospital in Toronto, Canada between 2016 and 2019 were included. Time-to-event analysis and Receiver Operating Characteristics Area Under the Curve (AUC) was conducted with the outcome variable being the occurrence of the first violent incident or first restraint event.

**Results:**

We included 3819 patients, of which 17% had at least one violent incident. We analysed 88,124 DASA scores and found a significant association with violence (HR 1.79 (95% CI), AUC 0.73). We found that the AUCs were similar for subspecialized forensic, schizophrenia and acute care units (0.71, 0.73 and 0.75 respectively), and lower for geriatric units (0.66). We propose new violence risk categories based on the frequency of violence at each score.

**Discussion:**

Higher DASA scores are associated with higher risk of violent incidents in both forensic and non-forensic inpatient psychiatric units. The proposed violence risk groups help rule out patients at low risk of violence and may help identify patients who would most benefit from interventions to reduce violence.

## Introduction

Violence in healthcare settings is a widespread problem which affects both patients and staff and costs an estimated 12.7 billion dollars annually in legal and healthcare costs in Canada alone (1). Over 60% of nurses in Canada reported violence in the workplace during the previous year (2), and as many as 70% of mental health staff report having been physically assaulted within the last 12 months (3). In acute psychiatric settings, around 20% of patients engage in violent behavior (4) and in forensic settings, as many as 30% to 40% of patients commit at least one act of violence within a one-year period (5).

Efforts to reduce violence include staff training (6), organizational policy and safety procedures and environmental design (7). Structured risk assessments can be used by clinicians to identify those at high risk for violence so that appropriate steps can be taken. Several risk assessment tools such as the Historical Clinical Risk Management tool (HCR-20) (8) and the Short-Term Assessment of Risk and Treatability tool (START) (9) have been shown to have good predictive validity. However, these tools are not ideally suited for inpatient settings, for the purpose of short-term monitoring of violence risk over the next hours to days (10).

Several short-term dynamic risk assessments have been developed to predict violence within a 24-hour window (11, 12), one of which is the Dynamic Appraisal of Situational Aggression (DASA). The DASA was originally developed in a secure forensic hospital in Melbourne, Australia. The DASA is a 7-item risk assessment tool that has been developed to assess short-term risk in adult forensic inpatient settings. The presence or absence of each of the seven items within the past 24-hours are totaled to give a score between zero and seven (13). Total DASA scores more accurately predict violence in multiple settings, as compared to unstructured clinical assessments (14–18), including among youth (19, 20), female forensic inpatients (21) and in the emergency department (22). More recently, Dickens and colleagues carried out a systematic review and meta-analysis of tools to assess imminent aggression, which included 14 patient samples that employed the DASA. The overall effect size (Hedge’s g coefficient) for prediction of interpersonal aggression and for any aggression was 1.04 [0.69, 1.39] and 0.88 [0.62, 1.15], respectively (23). To improve the utility of DASA, some efforts have been undertaken to optimize the way in which the score is analysed. One study showed that averaging the DASA score over a period of three days improved its predictive validity (24). A similar study by Chu and colleagues showed that averaging the DASA score may improve predictive validity (22).

Some studies have categorized the risk of violence into “low”, “medium” and “high” risk categories depending on the total DASA score. These categories can have practical value for clinical staff, who can mitigate risk by linking a clinical intervention to a risk severity category (25). This may have utility in resource prioritization and clinical interventions but there has been little investigation of the predictive validity of such categorization. However, there have been no studies that we are aware of that have directly compared the validity of the original risk categories in other settings. Examining different inpatient units would help determine the generalizability of the DASA across clinical settings – making the DASA a more universal tool for predicting short term risk of violence. Moreover, no studies have investigated alternative cut-offs for the risk categories. One limitation in the DASA tool has been the high sensitivity and low specificity of the original DASA cut-offs. We hypothesize that different clinical settings may benefit from different cut-off scores. For example, a chronic, long-term, psychiatric unit, with rare violent incidents, may wish to adjust the cut-off score to maximize specificity and minimize the number of false positives. Investigating different cut-offs, may help clinicians in determining the optimal use of the DASA for their clinical setting.

To date, there have been no large longitudinal studies that investigated the validity of DASA in predicting violent incidents during the first few weeks of a hospital admission. The largest sample to date included 1548 patients assessed within an emergency department in a single 24-hour period (22). Moreover, there have been no studies that compared validity across specialized and general psychiatric units in both forensic and non-forensic settings. We have therefore carried out a study to investigate the predictive validity of DASA among all newly admitted adult patients in a large mental health facility comprising acute, long-stay, elderly and forensic services. Our study aims are as follows: (1) to investigate the predictive validity of the DASA among new admissions in different populations; and (2) to investigate the predictive validity of alternative cut-off scores for risk categories.

## Methods

### Setting

We carried out this study at The Centre for Addiction and Mental Health (CAMH) in Toronto, Ontario, Canada’s largest mental health teaching hospital. The hospital has multiple units including acute care, forensic, geriatric and chronic schizophrenia programs. This study was carried out independently of the authors of the DASA.

### Patient population

We included all patients aged 18 years and over admitted to CAMH from 2016 to 2019. For those admitted more than once during that time, we included only the first admission. We excluded patients held only in the emergency department and those admitted for less than 24-hours. We also excluded those in which a violent incident occurred on the first day of admission (prior to a DASA being scored) because of the need to record DASA scores prior to a violent incident occurring and those with two or more consecutive missing DASA scores.

### Measures

The DASA comprises seven items that are recorded daily for every patient by nursing staff using observational and chart data from the last 24 hours. DASA produces a maximum score of 7 with each item scoring either a 0-1 (13). Violence risk was first classified as Low, Moderate, or High based on the standard cut off scores 0-1 , 2-3 and 4-7 respectively. Based on our preliminary investigation of predictive validity at alternative cut-off scores, we defined a “high specificity” categorization as Low (0-3), Medium (4-5) and high (6-7).

*The outcome measure* is the occurrence of the first violent incident following admission, which is defined as verbal or physical aggression towards a person or property. This definition is consistent with previous studies examining the validity of DASA (17, 18). Outcome measure data were collected from the electronic medical record (EMR) by identifying violent incidents reported in a SCORE report (Staff and Client On-line Reporting of Events- the hospital’s incident reporting system) or by the use of incidents of seclusion, mechanical restraint or chemical restraint where the documented reason was harm to others. It is hospital policy for all violent events to be recorded in the SCORE system. The use of seclusion or restraint events was to capture any violent outcome events that were not captured in SCORE.

### Clinical and demographic data

We collected clinical and demographic data including age, sex, DSM-5 diagnosis (26), legal status (voluntary, involuntary or informal) and unit type, categorized as adult acute care, geriatric, forensic and chronic schizophrenia units.

### Procedure and statistical analysis

The follow-up period for each patient was a maximum of 60 days, until discharge from hospital, or the first outcome incident, whichever came first. We restricted the analysis window to 60 days from the day of admission, adopting the methods used in a previous study (14), and because of the very low incidence of violence beyond this time.

Statistical analysis was conducted using the SAS Enterprise Guide version 7.1 software. The cohort was described by summarizing demographic data, DASA scores and the outcome measure using percentages and means where appropriate. The association between DASA score and outcome over a subsequent 24-hour period was measured.

Patients had DASA scores recorded daily, and therefore each DASA score was used as the predictor for violence in each subsequent 24-hour period. We used Cox proportional hazard regression, using DASA scores and violence risk groups, as time-dependent variables to model association with violence. We calculated the area under the Curve (AUC) for each 24-hour period and report the average AUCs. A difficulty in measuring the predictive validity of a violence risk assessment instrument in clinical settings is that a high score on the risk instrument would likely prompt a clinical intervention by healthcare staff to mitigate this risk, and thereby reducing the apparent association between risk instrument scores and outcome. Unscheduled medication (often referred to as Pro re nata (PRN) medication) is often given on an “as needed” basis, in inpatient settings aimed at reducing agitation or aggression. We therefore also included administration of unscheduled antipsychotic or benzodiazepine medication in the 24 hours prior to the incident as a time-varying variable in our model, to control for the effects of this intervention in reducing violence. Other clinical interventions with nursing staff which may have been prompted by a high DASA scores were not measured and therefore could not be included in the model.

We first investigated the association between the Low, Medium and High risk categories using the original cut-off scores reported by the authors (13), then repeated that analysis using the alternative “high specificity” cut-off scores to assign new risk categories. We then carried out the analyses separately for patients in forensic, schizophrenia, acute and geriatric units to calculate hazard ratios and AUC for each.

### Ethics statement

Research Ethical approval was granted by the Centre for Addictions and Mental Health Research Ethics Board (067/2020) for use of retrospective data for the purpose of research. No identifiable information was retained or is presented in this study.

## Results

There were 4,320 admissions during the study period. We excluded 501 patients (213 patients had a length of stay in hospital of less than 1 day, 16 patients had a violent event prior to a DASA score being recorded and 272 patients had 2 or more consecutive DASA scores missing) leaving 3819 patients included in the study. There were 88,124 DASA scores recorded. Demographic data, diagnosis, admission status and admission location for the study population are summarized in **Table 1**. The mean age of the patients studied was 39.5 (range 16-81); approximately two thirds were male. Just over half of patients had a diagnosis of schizophrenia spectrum or psychotic disorder and approximately one fifth of patients had a diagnosis of either bipolar and related disorders, substance-related and addictive disorders and depressive disorders. Around half of the patients were involuntarily admitted with the remainder being voluntarily admitted. The majority (84.6%) were admitted to an acute care unit, 8.1% were admitted to the geriatric psychiatry program, 6.2% admitted to the forensic program and 1.1% to the chronic schizophrenia program.

**Table 1:** Summary of demographic data for patients included in the study.

Characteristic	Value (% of total cohort)
Number of patients	3819
Mean age in years (range)	39.53 (18-81)
Gender	
Female	1354 (35.5%)
Male	2460 (64.4%)
Other	5 (0.1%)
Diagnosis	
Schizophrenia spectrum and psychotic disorders	2148 (56.2%)
Bipolar and related disorders	779 (20.4%)
Substance-related and addictive disorders	725 (19.0%)
Depressive disorder	689 (18.0%)
Neurocognitive disorder	311 (8.1%)
Personality disorder	278 (7.3%)
Missing diagnosis	206 (5.4%)
Other	581 (15.2%)
Admission Status	
Involuntary	1849 (48.4%)
Voluntary	1769 (46.3%)
Missing	201 (5.3%)
Admission Location	
Acute care program	3229 (84.6%)
Forensic program	237 (6.2%)
Geriatric psychiatry program	309 (8.1%)
Chronic Schizophrenia program	44 (1.1%)

The maximal DASA score reported during the study period is shown in **Table 2**. Almost one quarter of patients had a DASA score of zero through the period of observation, and 45% had a maximum score of 3 or more. Approximately 7-8% of patients had maximum scores of 5, 6 or 7. **Table 3** summarizes the time to outcome event for the study cohort. There were 634 patients who carried out at least one violent incident, representing 16.6% of the study cohort. We found that 82% of all outcome events (519 incidents) occurred in the first two weeks following admission. We found that 59% of patients categorized with a high DASA score received a PRN medication, 39% with a medium score and 14% with a low score for aggression or agitation.

**Table 2:** Summary of number and percentage of patients for each DASA peak score within the follow-up period of 60 days and time to outcome event.

DASA peak score	Number (%)
0	912 (23.9%)
1	680 (17.8%)
2	502 (13.1%)
3	484 (12.7%)
4	358 (9.4%)
5	291 (7.6%)
6	270 (7.1%)
7	321 (8.4%)

**Table 3:** Number and percentage of patients who were violent at each timeframe out of all patients per timeframe.

Time to outcome event (violence)	Number (%)
Days 1 to 3	217 (5.7%)
Days 4 to 7	172 (4.5%)
Week 2	130 (3.4%)
Week 3	52 (1.4%)
Week 4	25 (0.7%)
Week 5	10 (0.3%)
Week 6	12 (0.3%)
Week 7	10 (0.3%)
Week 8	5 (0.1%)
Week 9 (57 - 60 days)	1 (0.0%)

We found that DASA scores were significantly associated with violence overall (HR 1.79, CI 1.73-1.85 (per unit increase)). The overall AUC was 0.73. We investigated two alternative groupings of Low, Medium and High risk of violence with either high sensitivity or high specificity. The high sensitivity groupings are as follows: scores 0-1, 2-3 and 4-7 for Low, Moderate and High risk of violence. The high specificity grouping had the following categorization: scores of 0-3, 4-5 and 6-7 for Low, Moderate and High risk of violence. Both groupings were analysed using the Cox proportional hazard model (see **Table 4**). The high sensitivity grouping hazard ratio comparing medium and low risk groups was 5.38 (CI 4.38-6.61) and the hazard ratio comparing low to high-risk group was 18.98 (CI 15.69-22.97). For the high specificity groupings, the hazard ratios comparing medium and low risk groups was 8.4 (CI 6.87-10.28) and the hazard ratio comparing low to high-risk group was 20.3 (CI 16.41-25.12) (see **Table 4**).

**Table 4:** Hazard ratios for either moderate or high violence risk categories for DASA across different inpatient psychiatric units.

	Incidents	No incidents	Hazard Ratio	p
High Sensitivity Group				
Low (0-1)	217 (0.31)	69417 (99.69)	Reference	
Medium (2-3)	172 (1.46)	11575 (98.53)	5.38 (4.38-6.61)	< 0.001
High (4-7)	245 (3.63)	6498 (96.37)	18.98 (15.69-22.97)	< 0.001
High Specificity Group				
Low (0-3)	306 (0.40)	76462 (99.60)	Reference	
Medium (4-5)	143 (3.13)	4416 (96.86)	8.40 (6.87-10.28)	< 0.001
High (6-7)	102 (4.67)	2082 (95.32)	20.30 (16.41-25.12)	< 0.001

Finally, we compared the performance of the DASA in predicting violent events between the different specialized units at CAMH (See **Table 5**). We found that the AUCs were similar for forensic, schizophrenia and acute care units (0.71, 0.73 and 0.75 respectively), and lower for geriatric units (0.66). We found that hazard ratios were similar across units with lowest on forensic units (HR 1.44, CI 1.31-1.59) and highest on geriatric units (HR 2.02, CI 1.78-2.29).

**Table 5:** DASA score validity compared between different specialized units.

Unit	Days with Violence	Days without Violence	Hazard Ratio	p	AUC
Forensic	91	11,471	1.44 (1.31-1.59)	<0.001	0.71
Schizophrenia	105	15,500	1.85 (1.69.2.02)	<0.001	0.73
Acute Care	372	45,518	1.83 (1.76-1.91)	<0.001	0.75
Geriatric	50	11,615	2.02(1.78-2.29)	<0.001	0.66

## Discussion

In our study of 3819 patients admitted to a large mental health facility, we found that the DASA was a moderately good predictor of violent incidents overall. We also found that although the DASA performed well in all settings, the predictive accuracy was lower on geriatric units in comparison to acute, schizophrenia and forensic units, and we found that alternative risk categories to those originally defined may have better performance. This has clinical implications for aligning resources with predicted need in efforts to reduce violence on in-patient settings.

Our results are consistent with previous literature that has validated the DASA (13–15, 17, 18). Our study similarly found that higher DASA scores are significantly associated with higher likelihood of violence, and additionally found that this held across a diverse patient population.

The main objective of our study was to examine the validity of the DASA instrument in a large inpatient population across different units. The original DASA authors proposed risk categories that were compared to unit staff’s clinical judgement (13). We explored alternative cut-off scores for categorizing Low, Moderate and High risk groups that may have practical and clinical implications in healthcare settings.

We found that 82% of first violent incidents occur in the first two weeks of admission. Patients are likely more acutely ill at the start of their admission and are more likely to be involved in violent incidents. Patients that are at a low risk of violence based on the DASA score in these first two weeks are unlikely to have a violent incident during their admission. This finding, based on our high specificity risk categories, can allow psychiatric units to identify patients in need of more intensive risk management strategies and to intervene early in the course of an admission. Although there is a significant association between higher DASA scores and violence, the AUCs are moderate which are consistent with other tools that assess short term risk such as the Broset Violence Checklist (AUC = 0.69) (27). In our study, even the highest scores on the DASA resulted in a violent incident in only around 5% of occasions.

The DASA score has been incorporated into an aggression prevention protocol to better help staff manage risk of violence (28) and provide guidance around situations requiring staff intervention such as verbal de-escalation or pharmacotherapy (29). Given limited healthcare resources and low proportion of violent incidents, a strategy directing resources to high-risk individuals is ideal, where the need is greatest (30). We selected violence risk categories that result in high specificity to identify more individuals that are of low risk of violence where fewer resources are needed. This comparison was ideally suited for our large sample size where we were able to ensure DASA remains valid across multiple violence risk categories and across multiple inpatient units.

Overall there was little difference in the performance of the DASA across different units, however the predictive accuracy was slightly lower on geriatric units. The majority of the patients on geriatic units had neurocognitive disorders, and therefore the items of the DASA may not be as relevant in predicting aggression compared with those on other units, where the primary diagnoses are psychotic illnesses.

A major strength of our study is the large sample, gathering an almost complete cohort of new admissions over a two-year period, which to our knowledge is the largest study analyzing the DASA to date. This allowed our study to investigate the performance of the DASA across multiple specialized inpatient units in both forensic and non-forensic settings making our findings applicable across a broad setting. We were also able to offer clinical implications while analyzing different violence risk categories.

There are however a number of limitations of our study. First, our study only included the first violent incident, and therefore our findings may not apply to the predictive validity of DASA score in the context of repeat violent incidents during a single admission. This does however eliminate previous violent incidents as a confounder which has been shown to be a significant variable in predicting violence (31). Second, we did not have any data to assess clinical interventions that may be differentially applied to those identified as high risk, other than PRN medication administration. The association between higher DASA scores and violence is therefore underestimated due to likely clinical interventions to mitigate risk of violence that are not captured by our study. Third, our study is retrospective, and it is difficult to control for all potential biases in our cohort, such as the reliability of DASA scores recorded by all staff.

For future research it will be important to address the question of whether our findings can guide intervention for violence prevention and how this may impact the incidence of violence in the inpatient setting. It also remains unclear how a violent incident during an admission changes the validity of the DASA. Multiple risk factors that correlate with risk of violence have been identified in previous literature (4, 31, 32) but it remains unknown whether a comprehensive model that includes clinical data, risk factors and the DASA may improve violence prediction.

## Conclusions

Our large-scale longitudinal patient dataset confirmed that the DASA has moderate predictive validity for predicting risk of violence in inpatient units. The DASA is valid across both forensic and non-forensic units, though further work is needed to establish the utility on geriatric units. We have proposed new violence risk categories for low, moderate, and high risk of violence, which may help improve the clinical utility of the DASA score in directing intervention to those most at risk. Our study also found that patients who are consistently at low risk of violence based on the DASA score, for the first two weeks of admission, are likely to remain at a low risk and unlikely to be involved in a violent incident. Further research is needed to look at how DASA can guide interventions and whether combining risk factors and clinical data with the DASA score improves its predictive power.

## Data Availability

The raw data supporting the conclusions of this article will be made available by the authors, without undue reservation.
